# Association between lipoprotein(a), fibrinogen and their combination with all-cause, cardiovascular disease and cancer-related mortality: findings from the NHANES

**DOI:** 10.1186/s12889-024-19443-4

**Published:** 2024-07-18

**Authors:** Zhenwei Wang, Xuejiao Yan, Lijuan Fang, Junnan Tang, Jinying Zhang

**Affiliations:** 1 Department of Cardiology, The First Affiliated Hospital of Zhengzhou University, Zhengzhou, 450000 China; 2 Key Laboratory of Cardiac Injury and Repair of Henan Province, Zhengzhou, 450018 China; 3 Henan Province Clinical Research Center for Cardiovascular Diseases, Zhengzhou, 450052 China; 4 Department of Cardiology, The Affiliated Changzhou No.2 People’s Hospital of Nanjing Medical University, Changzhou, 213003 China; 5 Department of Cardiology, The First Hospital of Hohhot, Hohhot, 010030 China

**Keywords:** Lipoprotein(a), Fibrinogen, Mortality, Cardiovascular mortality, Cancer mortality

## Abstract

**Background:**

There is evidence indicating that both lipoprotein(a) [Lp(a)] and fibrinogen (FIB) are associated with mortality, However, the impact of their combination on mortality has not been determined. Thus, the aim of this study was to examine the association between the combination of Lp(a) and FIB with all-cause and cause-specific mortality.

**Methods:**

This prospective cohort study enrolled 4,730 participants from the third National Health and Nutrition Examination Survey. The exposure variables included Lp(a), FIB and their combination, while the outcome variables consisted of all-cause, cardiovascular disease (CVD) and cancer-related mortality. Multivariate COX regression, subgroup analysis, sensitivity analysis and restricted cubic spline (RCS) were used to investigate the association between Lp(a), FIB and their combination with all-cause, CVD and cancer-related mortality.

**Results:**

Over a median follow-up period of 235 months, 2,668 individuals died, including 1,051 deaths attributed to CVD and 549 deaths due to cancer. Multivariate Cox regression analyses revealed independent associations between both Lp(a) and FIB with all-cause, CVD, and cancer-related mortality. Compared to participants in the 1st to 50th percentiles of both Lp(a) and FIB, those in the 90th to 100th percentiles exhibited multivariable adjusted HRs of 1.813 (95% CI: 1.419–2.317, *P* < 0.001), 2.147 (95% CI: 1.483–3.109, *P* < 0.001) and 2.355 (95% CI: 1.396, 3.973, *P* = 0.001) for all-cause, CVD and cancer-related mortality, respectively. Subgroup and sensitivity analyses did not substantially attenuate the association between the combination of high Lp(a) and high FIB with the risk of all-cause and CVD-related mortality. Additionally, the RCS analysis showed that the relationship between Lp(a) and the risk of all-cause and cancer-related mortality, as well as the relationship between FIB and the risk of cancer-related mortality, were linear (P for nonlinearity > 0.05). Conversely, the relationship between Lp(a) and the risk of CVD-related mortality, as well as the relationship between FIB and the risk of all-cause and CVD-related mortality, were nonlinear (P for nonlinearity < 0.05).

**Conclusions:**

High levels of Lp(a) and FIB together conferred a greater risk of mortality from all-cause, CVD and cancer.

## Introduction

The Global Burden of Diseases (GBD) Collaborators have provided data on mortality rates for 282 causes of death across 195 countries and regions from 1980 to 2017 [1]. In 2017, the worldwide death toll reached 55.9 million, with chronic non-communicable diseases being responsible for the majority of deaths at approximately 41.1 million (73.4%) [1]. Among these diseases, three chronic non-communicable diseases causing the most deaths were cardiovascular disease (CVD) (17.8 million), cancer (9.56 million) and chronic respiratory diseases (3.91 million), respectively [1]. Thus, it is crucial to identify preventable and manageable risk factors in order to address this situation. While smoking, hypertension and diabetes have been established as controllable independent risk factors for premature death, there still exists a considerable number of unexplained deaths.

There is evidence indicating that higher levels of lipoprotein(a) [Lp(a)] are not only causally associated with a higher prevalence of CVD, but they may also be associated with all-cause, CVD, and cancer-related mortality [2, 3]. For example, Fogacci et al. found that Lp(a) was an independent predictor of CVD-related mortality in individuals at high cardiovascular risk, as well as in women at intermediate risk [4]. In addition, in a large prospective cohort study, Langsted et al. also found that higher Lp(a) levels were independently associated with higher all-cause mortality and CVD-related mortality [5]. However, two other studies demonstrated that there was no statistically significant association between Lp(a) and all-cause mortality, CVD-related mortality, cancer-related mortality, or non-vascular mortality [6, 7]. Therefore, it can be observed that there is no consensus regarding the association between Lp(a) and mortality. This lack of agreement may be attributed to variations in the level of involvement of Lp(a) in the pathogenesis. Current evidence suggests that Lp(a) is a low-density lipoprotein-like particle covalently bound to apolipoprotein(a) [apo(a)] by apolipoprotein B (apoB) through a single disulfide bond, with apo(a) originating from the fibrinogen (FIB) gene through replication and remodeling, so the pathogenic effects of Lp(a) mainly include pro-atherogenic and pro-thrombotic properties [8–10]. Unlike apoB, apo(a) does not contain a lipid domain and is not involved in lipid transportation. On the contrary, it can promote thrombosis and potentially produce an antifibrinolytic effect by inhibiting the activation of plasminogen [11]. Additionally, there is evidence suggesting that the impact of high Lp(a) on mortality is greater than what can be explained by its cholesterol content [5]. Therefore, the effect of Lp(a) on mortality likely involves the fibrinolytic system. As an important component of the fibrinolytic system, plasma FIB has been well established as an independent risk factor for cardiovascular events and mortality [12–17].

However, it remains unknown whether the combination of extremely high levels of Lp(a) and FIB was associated with the highest risk of mortality. Therefore, to address this knowledge gap, our study aimed to explore the association between the combination of Lp(a) and FIB with all-cause and cause-specific mortality.

## Materials and methods

### Study population

**Fig. 1:**
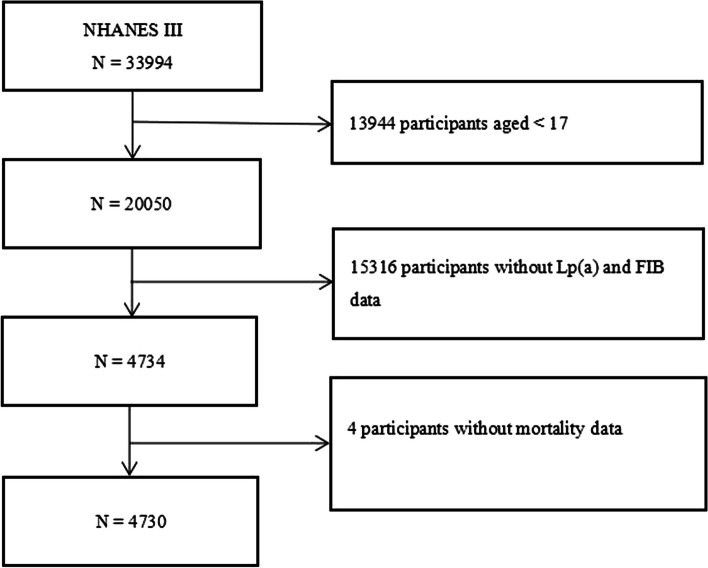
Flow chart of the study population. NHANES III, the third National Health and Nutrition Examination Survey; Lp(a), lipoprotein (a); FIB, fibrinogen

The National Health and Nutrition Examination Survey (NHANES) is a national survey that observes the health and nutrition of adults and children in the United States, and it is distinct as it combines interviews and physical exams, and it is managed by the National Center for Health Statistics (NCHS), a division of the Centers for Disease Control and Prevention (CDC), which is in charge of producing important health statistics for the country [18]. The third NHANES (NHANES III), a nationwide survey conducted from 1988 to 1994 in two phases, consisted of a probability sample of 39,695 individuals aged 2 months and older, with both phases and the combined six-year period offering nationally representative samples [19, 20]. This study included 4,730 participants who were selected from the NHANES III after excluding minors and individuals without Lp(a), FIB and mortality data (Fig. 1). The NHANES III survey protocol was approved by the NCHS of the CDC Institutional Review Board. All participants provided written informed consent when participating in NHANES III, and this study adhered to the Declaration of Helsinki.

### Covariates collection and definitions

All the data and information were downloaded from the NHANES official website (https://www.cdc.gov/nchs/nhanes/). The covariates analyzed in this study included age, sex, race, education, marital status, family poverty income ratio (PIR), ideal exercise, smoking status, drinking, CVD, diabetes, hypertension, hypercholesterolemia, cancer, hypotensive drugs, hypoglycemic drugs, cholesterol-lowering drugs, body mass index (BMI), systolic blood pressure (SBP), diastolic blood pressure (DBP), total cholesterol (TC), triglycerides (TG), low-density lipoprotein cholesterol (LDL-C), high-density lipoprotein cholesterol (HDL-C), blood urea nitrogen (BUN), creatinine (CR), uric acid (UA), fasting plasma glucose (FPG) and hemoglobin A1c (HbA1c). The aforementioned demographic data were obtained through a standard household interview questionnaire, while anthropometric data were measured by professionals through standard screening procedures. Comorbidity and medication data were obtained from self-reported information in the household interview questionnaire. Blood markers were measured or estimated by trained professionals through standard and validated biochemical analysis procedures. The quality control of the laboratory components of the NHANES can be found in published literature [21]. In our study, we divided race into four groups: non-Hispanic White, non-Hispanic Black, Mexican American and Others. Family PIR was divided into three groups based on thresholds of 1.0 and 3.0: ≤ 1.0, 1.0–3.0, and > 3.0. Ideal exercise was defined as engaging in at least 75 min of high-intensity exercise or at least 150 min of moderate-intensity exercise per week [22]. Smoking status was categorized into three groups based on the smoking habit of the individual: not at all, some days and every day. Alcohol consumption was defined as having consumed at least 12 drinks in the last one year. BMI was calculated by dividing weight in kilograms by the square of height in meters. CVD was defined as the presence of coronary heart disease, heart attack, stroke or congestive heart failure. Hypertension was diagnosed if SBP ≥ 140 mmHg or DBP ≥ 90 mmHg and information on comorbidities and medication use from the household interview questionnaire indicated its presence, where the values for SBP and DBP were the average of three consecutive blood pressure readings [23]. Diabetes was diagnosed if FPG ≥ 7.0 mmol/L or HbA1c ≥ 6.5%, with information on comorbidities and medication use obtained from the household interview questionnaire [24]. Cancer was diagnosed based on information on comorbidities from the household interview questionnaire, encompassing all types of cancer recorded in NHANES.

### Measurement of Lp(a) and FIB

The concentrations of Lp(a) and FIB were determined in this study using serum and plasma samples, respectively. Trained laboratory staff, following standard protocols, employed enzyme-linked immunosorbent assay to measure apo(a) levels and enzyme assay to measure plasma FIB levels. The results were reported in g/L according to the international system of units. Further information can be found elsewhere [25, 26].

### Follow-up and outcomes

The prognostic data of all participants were obtained by matching NHANES with the National Death Index, including follow-up time and mortality data. In this study, all-cause mortality, CVD-related mortality, and cancer-related mortality, as diagnosed according to ICD-10 codes, were identified as the outcome variables [27]. All participants were followed up from the date of their household interview until the occurrence of the outcome variables or December 31, 2015.

### Statistical analysis

Due to the nature of the multi-stage probability sampling design of NHANES, we adjusted the weights in our analysis to avoid oversampling and reduce the nonresponse rate. Specifically, data for continuous and categorical variables were expressed as weighted means (95% CIs) and weighted percentages (95% CIs), respectively. To explore the relationship between Lp(a) and FIB levels and mortality in more detail, we followed the unconventional percentile grouping method of Kaltoft et al. and divided all participants into low (L), medium (M) and high (H) groups according to the percentile of Lp(a): 1–50 Percentiles (0.18 g/L), 51–89 Percentiles (0.19–0.66 g/L) and 90–100 Percentiles (≥ 0.67 g/L) [28]. We evaluated the differences in continuous or categorical variables among different Lp(a) groups using weighted linear regression or the weighted Chi-square test, respectively. Similarly, participants were divided into L, M and H groups according to the percentile of FIB: 1–50 Percentiles (≤ 3.05 g/L), 51–89 Percentiles (3.06–4.09 g/L) and 90–100 Percentiles (≥ 4.10 g/L). Likewise, differences between the FIB groups were assessed using weighted linear regression (for continuous variables) or weighted Chi-square test (for categorical variables). Then, we created nine groups by combining the three Lp(a) and FIB groups in two-by-two combinations: Lp(a)-L + FIB-L, Lp(a)-L + FIB-M, Lp(a)- L + FIB-H, Lp(a)-M + FIB-L, Lp(a)-M + FIB-M, Lp(a)-M + FIB -H, Lp(a)-H + FIB-L, Lp(a)-H + FIB-M, and Lp(a)-H + FIB-H. Two multivariate Cox proportional hazard models were constructed to explore the associations of Lp(a), FIB, and their combination with all-cause, CVD, and cancer mortality. In this study, the variables selected for adjustment in the multivariable models were based on univariate Cox regression analyses to control for known confounders. We adjusted for covariates related to mortality (*P* < 0.05) from the univariate Cox regression analysis, including age and sex. Survival probabilities between groups were evaluated using the Kaplan–Meier method, with differences compared using the log-rank test. Subgroup analysis was performed according to sex, excluding sex from the multivariate model. A sensitivity analysis was also conducted by excluding patients who died within two years of follow-up. Finally, restricted cubic spline (RCS) analysis assessed potential nonlinear associations between Lp(a) and FIB with mortality outcomes. All analyses were performed using SPSS 26.0 and R 4.1.3, with a two-tailed *P* value < 0.05 considered statistically significant.

## Results

### Baseline characteristics

As shown in Table 1, the differences in race, education, marital status, hypercholesterolemia, CVD, cholesterol-lowering drugs, BMI, DBP, TG, TC, LDL-C, FIB and FPG among the three groups of Lp(a) were statistically significant (*P* < 0.05). Participants with higher Lp(a) had higher levels of FIB than participants with lower Lp(a) (*P* < 0.001). Similarly, as observed in Table 2, variables other than hypercholesterolemia, DBP, TG and UA were statistically significant among the three groups of FIB. And participants with higher FIB had higher levels of Lp(a) than participants with lower FIB (*P* < 0.001).

**Table 1:** Baseline characteristics of participants stratified by the Lp(a)

	**Total population**	**Lp(a)**			***P* value**
		**1–50 Percentiles**	**51–89 Percentiles**	**90–100 Percentiles**	
		** ≤ 0.18 g/L**	**0.19–0.66 g/L**	** ≥ 0.67 g/L**	
Age, years	57.35 (56.05, 58.64)	57.20 (55.70, 58.71)	57.34 (55.92, 58.76)	58.38 (57.31, 59.44)	0.373
Sex, male, n (%)	46.37 (43.98, 48.79)	47.53 (44.30, 50.78)	45.32 (41.82, 48.86)	42.73 (36.69, 48.99)	0.281
Race, n (%)					< 0.001
Non-Hispanic White	79.94 (75.58, 83.68)	85.16 (81.41, 88.26)	76.39 (70.65, 81.31)	58.11 (48.02, 67.56)	
Non-Hispanic Black	9.20 (7.53, 11.20)	3.37 (2.59, 4.37)	13.64 (11.25, 16.45)	31.40 (24.16, 39.67)	
Mexican–American	3.64 (2.89, 4.58)	4.37 (3.52, 5.43)	2.93 (2.24, 3.82)	1.52 (0.96, 2.40)	
Others	7.22 (4.62, 11.12)	7.10 (4.62, 10.75)	7.03 (3.83, 12.58)	8.98 (4.09, 18.56)	
Education					0.026
Less than high school	19.18 (15.99, 22.84)	18.18 (15.27, 21.50)	19.46 (15.75, 23.81)	25.20 (19.06, 32.53)	
High school or equivalent	41.35 (39.12, 43.62)	43.15 (39.44, 46.94)	38.45 (35.20, 41.80)	41.34 (35.30, 47.64)	
Higher than high school	39.47 (35.16, 43.95)	38.67 (33.66, 43.94)	42.09 (36.71, 47.66)	33.46 (26.23, 41.56)	
Marital status					0.002
Married	68.33 (65.39, 71.14)	70.89 (67.28, 74.25)	65.44 (61.45, 69.23)	62.77 (57.28, 67.95)	
Non-married	31.67 (28.86, 34.61)	29.11 (25.75, 32.72)	34.56 (30.77, 38.55)	37.23 (32.05, 42.72)	
Family PIR, n (%)					0.025
≤ 1.0	9.14 (6.44, 12.80)	8.04 (5.41, 11.79)	10.15 (6.77, 14.95)	12.64 (8.81, 17.82)	
1.0–3.0	39.31 (34.98, 43.82)	38.63 (33.66, 43.85)	39.01 (34.22, 44.02)	45.61 (37.31, 54.16)	
> 3.0	51.55 (45.19, 57.86)	53.33 (46.49, 60.04)	50.84 (43.94, 57.71)	41.75 (33.49, 50.49)	
Ideal exercise, n (%)					0.940
Yes	61.08 (56.54, 65.43)	60.79 (55.84, 65.53)	61.19 (53.98, 67.94)	62.61 (54.24, 70.28)	
No	38.92 (34.57, 43.46)	39.21 (34.47, 44.16)	38.81 (32.06, 46.02)	37.39 (29.72, 45.76)	
Smoking status, n (%)					0.124
Every day	44.57 (42.46, 46.70)	42.54 (39.80, 45.32)	48.37 (43.90, 52.88)	42.31 (34.57, 50.44)	
Some days	34.08 (31.46, 36.80)	36.56 (33.16, 40.10)	30.17 (24.53, 36.49)	33.62 (27.52, 40.32)	
Not at all	21.35 (19.21, 23.65)	20.90 (18.34, 23.72)	21.45 (17.58, 25.91)	24.07 (19.32, 29.56)	
Drinking, n (%)					0.284
Yes	52.04 (47.49, 56.56)	53.30 (48.56, 57.98)	49.80 (43.61, 55.99)	52.89 (45.95, 59.72)	
No	47.96 (43.44, 52.51)	46.70 (42.02, 51.44)	50.20 (44.01, 56.39)	47.11 (40.28, 54.05)	
Comorbidities, n (%)					
Hypertension					0.143
Yes	45.49 (42.17, 48.86)	44.32 (39.95, 48.79)	45.66 (41.41, 49.97)	53.20 (44.68, 61.54)	
No	54.51 (51.14, 57.83)	55.68 (51.21, 60.05)	54.34 (50.03, 58.59)	46.80 (38.46, 55.32)	
Diabetes					0.396
Yes	18.68 (16.89, 20.61)	19.53 (17.15, 22.16)	17.15 (14.29, 20.44)	19.39 (14.47, 25.50)	
No	81.32 (79.39, 83.11)	80.47 (77.84, 82.85)	82.85 (79.56, 85.71)	80.61 (74.50, 85.53)	
Hypercholesterolemia					< 0.001
Yes	42.59 (39.22, 46.04)	41.54 (37.44, 45.76)	40.96 (37.08, 44.95)	57.51 (49.96, 64.71)	
No	57.41 (53.96, 60.78)	58.46 (54.24, 62.56)	59.04 (55.05, 62.92)	42.49 (35.29, 50.04)	
CVD					0.004
Yes	10.12 (8.72, 11.72)	9.56 (7.91, 11.51)	9.65 (8.18, 11.35)	16.29 (11.24, 23.04)	
No	89.88 (88.28, 91.28)	90.44 (88.49, 92.09)	90.35 (88.65, 91.82)	83.71 (76.96, 88.76)	
Cancer					0.119
Yes	12.30 (10.71, 14.08)	13.19 (10.86, 15.92)	11.77 (9.95, 13.89)	8.23 (5.15, 12.90)	
No	87.70 (85.92, 89.29)	86.81 (84.08, 89.14)	88.23 (86.11, 90.05)	91.77 (87.10, 94.85)	
Treatment, n (%)					
Hypotensive drugs					0.369
Yes	23.62 (21.70, 25.66)	22.94 (19.91, 26.27)	23.78 (20.97, 26.84)	27.93 (22.30, 34.36)	
No	76.38 (74.34, 78.30)	77.06 (73.73, 80.09)	76.22 (73.16, 79.03)	72.07 (65.64, 77.70)	
Hypoglycemic drugs					0.242
Yes	6.32 (5.41, 7.37)	6.46 (5.13, 8.10)	5.67 (4.37, 7.32)	8.25 (6.35, 10.64)	
No	93.68 (92.63, 94.59)	93.54 (91.90, 94.87)	94.33 (92.68, 95.63)	91.75 (89.36, 93.65)	
Cholesterol-lowering drugs					< 0.001
Yes	10.75 (8.02, 14.25)	9.68 (7.10, 13.07)	9.91 (6.74, 14.35)	23.57 (16.38, 32.67)	
No	89.25 (85.75, 91.98)	90.32 (86.93, 92.90)	90.09 (85.65, 93.26)	76.43 (67.33, 83.62)	
BMI, kg/m^2^	27.53 (27.17, 27.89)	27.79 (27.34, 28.24)	27.08 (26.70, 27.46)	27.72 (27.10, 28.34)	0.017
SBP, mmHg	129.92 (128.90, 130.93)	129.66 (128.12, 131.19)	130.04 (128.65, 131.42)	131.28 (128.93, 133.62)	0.549
DBP, mmHg	76.27 (75.47, 77.07)	75.87 (75.01, 76.72)	76.83 (76.06, 77.60)	76.73 (74.86, 78.60)	0.019
TG, mmol/L	1.88 (1.82, 1.93)	2.05 (1.95, 2.15)	1.65 (1.59, 1.71)	1.64 (1.52, 1.76)	< 0.001
TC, mmol/L	5.58 (5.53, 5.64)	5.49 (5.43, 5.56)	5.62 (5.54, 5.70)	6.08 (5.93, 6.23)	< 0.001
LDL‑C, mmol/L	3.51 (3.45, 3.57)	3.39 (3.32, 3.46)	3.60 (3.50, 3.69)	3.88 (3.64, 4.12)	0.003
HDL‑C, mmol/L	1.29 (1.27, 1.32)	1.27 (1.24, 1.31)	1.32 (1.28, 1.35)	1.34 (1.27, 1.40)	0.114
FIB, g/L	3.05 (2.99, 3.12)	3.00 (2.92, 3.08)	3.08 (3.03, 3.13)	3.32 (3.21, 3.43)	< 0.001
BUN, mmol/L	5.52 (5.40, 5.64)	5.51 (5.37, 5.64)	5.53 (5.38, 5.68)	5.57 (5.24, 5.91)	0.914
CR, umol/L	98.62 (97.71, 99.54)	98.47 (97.38, 99.55)	98.59 (97.33, 99.86)	99.88 (96.54, 103.23)	0.738
UA, umol/L	327.19 (322.43, 331.96)	329.69 (324.10, 335.28)	322.49 (316.48, 328.49)	330.12 (315.08, 345.15)	0.164
FPG, mmol/L	5.80 (5.69, 5.90)	5.88 (5.72, 6.03)	5.64 (5.55, 5.72)	5.91 (5.70, 6.11)	0.004
HbA1c, %	5.66 (5.60, 5.72)	5.67 (5.60, 5.75)	5.61 (5.52, 5.70)	5.82 (5.69, 5.94)	0.059
Outcomes, n (%)					
All-cause mortality					0.770
Yes	44.25 (40.95, 47.60)	43.85 (39.14, 48.67)	44.46 (41.59, 47.37)	46.17 (41.59, 50.82)	
No	55.75 (52.40, 59.05)	56.15 (51.33, 60.86)	55.54 (52.63, 58.41)	53.83 (49.18, 58.41)	
CVD-related mortality					0.264
Yes	15.64 (13.86, 17.60)	14.79 (12.18, 17.86)	16.54 (14.52, 18.79)	17.69 (15.23, 20.46)	
No	84.36 (82.40, 86.14)	85.21 (82.14, 87.82)	83.46 (81.21, 85.48)	82.31 (79.54, 84.77)	
Cancer-related mortality					0.799
Yes	9.54 (8.33, 10.89)	9.22 (7.70, 11.02)	9.82 (7.82, 12.25)	10.52 (6.87, 15.79)	
No	90.46 (89.11, 91.67)	90.78 (88.98, 92.30)	90.18 (87.75, 92.18)	89.48 (84.21, 93.13)	

Data were expressed as weighted mean (95% CI), or weighted percentage (95% CI)

*Abbreviation*: *Lp(a)* Lipoprotein (a), *PIR* Poverty income ratio, *CVD* Cardiovascular disease, *BMI* Body mass index, *SBP* Systolic blood pressure, *DBP* Diastolic blood pressure, *TG* Triglycerides, *TC* Total cholesterol, *LDL-C* Low-density lipoprotein cholesterol, *HDL-C* High-density lipoprotein cholesterol, *FIB* Fibrinogen, *BUN* Blood urea nitrogen, *CR* Creatinine, *UA* Uric acid, *FPG* Fasting plasma glucose, *HbA1c* Hemoglobin A1c, *CI* Confidence interval

**Table 2:** Baseline characteristics of participants stratified by the FIB

	**FIB**			***P* value**
	**1–50 Percentiles**	**51–89 Percentiles**	**90–100 Percentiles**	
	** ≤ 3.05 g/L**	**3.06–4.09 g/L**	** ≥ 4.10 g/L**	
Age, years	55.36 (54.16, 56.57)	59.69 (57.77, 61.62)	61.49 (59.19, 63.80)	< 0.001
Sex, male, n (%)	50.09 (46.08, 54.10)	40.71 (37.51, 43.98)	44.23 (36.27, 52.49)	0.002
Race, n (%)				0.009
Non-Hispanic White	81.48 (77.09, 85.20)	78.92 (73.95, 83.16)	73.03 (64.20, 80.35)	
Non-Hispanic Black	8.19 (6.46, 10.33)	9.69 (7.76, 12.05)	14.48 (11.29, 18.39)	
Mexican–American	3.60 (2.76, 4.69)	3.75 (2.97, 4.72)	3.44 (2.27, 5.17)	
Others	6.73 (4.44, 10.07)	7.63 (4.37, 13.01)	9.05 (4.71, 16.69)	
Education				< 0.001
Less than high school	16.05 (13.43, 19.06)	21.51 (16.94, 26.91)	31.87 (26.30, 38.00)	
High school or equivalent	38.60 (35.97, 41.30)	46.39 (41.52, 51.34)	39.05 (33.55, 44.85)	
Higher than high school	45.35 (41.27, 49.50)	32.10 (26.90, 37.79)	29.08 (21.78, 37.65)	
Marital status				0.013
Married	70.94 (67.02, 74.56)	66.39 (62.13, 70.39)	57.82 (47.13, 67.83)	
Non-married	29.06 (25.44, 32.98)	33.61 (29.61, 37.87)	42.18 (32.17, 52.87)	
Family PIR, n (%)				< 0.001
≤ 1.0	6.74 (4.77, 9.45)	11.96 (7.89, 17.73)	14.52 (9.27, 22.02)	
1.0–3.0	36.64 (31.02, 42.65)	43.20 (38.58, 47.94)	41.94 (33.97, 50.35)	
> 3.0	56.62 (49.95, 63.05)	44.84 (37.28, 52.65)	43.54 (33.21, 54.47)	
Ideal exercise, n (%)				< 0.001
Yes	58.03 (52.97, 62.93)	64.34 (59.16, 69.19)	68.98 (61.87, 75.29)	
No	41.97 (37.07, 47.03)	35.66 (30.81, 40.84)	31.02 (24.71, 38.13)	
Smoking status, n (%)				0.002
Every day	47.70 (43.92, 51.50)	40.31 (36.09, 44.69)	40.54 (34.09, 47.33)	
Some days	34.51 (30.97, 38.23)	33.51 (29.80, 37.43)	33.46 (27.63, 39.85)	
Not at all	17.79 (14.90, 21.10)	26.18 (22.49, 30.24)	26.00 (18.24, 35.63)	
Drinking, n (%)				< 0.001
Yes	58.46 (52.99, 63.73)	44.99 (40.28, 49.78)	36.06 (29.36, 43.36)	
No	41.54 (36.27, 47.01)	55.01 (50.22, 59.72)	63.94 (56.64, 70.64)	
Comorbidities, n (%)				
Hypertension				< 0.001
Yes	38.80 (34.75, 43.02)	54.55 (50.42, 58.62)	54.47 (45.57, 63.09)	
No	61.20 (56.98, 65.25)	45.45 (41.38, 49.58)	45.53 (36.91, 54.43)	
Diabetes				< 0.001
Yes	15.56 (13.35, 18.05)	21.46 (18.52, 24.72)	29.38 (26.20, 32.77)	
No	84.44 (81.95, 86.65)	78.54 (75.28, 81.48)	70.62 (67.23, 73.80)	
Hypercholesterolemia				0.226
Yes	40.68 (36.01, 45.52)	44.99 (40.77, 49.28)	46.55 (37.14, 56.21)	
No	59.32 (54.48, 63.99)	55.01 (50.72, 59.23)	53.45 (43.79, 62.86)	
CVD				< 0.001
Yes	6.39 (5.13, 7.93)	13.35 (10.98, 16.13)	23.26 (17.53, 30.17)	
No	93.61 (92.07, 94.87)	86.65 (83.87, 89.02)	76.74 (69.83, 82.47)	
Cancer				0.006
Yes	10.17 (8.37, 12.30)	15.46 (12.58, 18.85)	13.89 (8.91, 21.01)	
No	89.83 (87.70, 91.63)	84.54 (81.15, 87.42)	86.11 (78.99, 91.09)	
Treatment, n (%)				
Hypotensive drugs				< 0.001
Yes	19.01 (17.03, 21.16)	28.64 (24.95, 32.64)	36.00 (28.74, 43.97)	
No	80.99 (78.84, 82.97)	71.36 (67.36, 75.05)	64.00 (56.03, 71.26)	
Hypoglycemic drugs				< 0.001
Yes	3.92 (3.10, 4.94)	8.00 (6.46, 9.87)	16.55 (13.15, 20.63)	
No	96.08 (95.06, 96.90)	92.00 (90.13, 93.54)	83.45 (79.37, 86.85)	
Cholesterol-lowering drugs				< 0.001
Yes	7.99 (5.33, 11.81)	13.46 (9.80, 18.21)	19.50 (12.09, 29.89)	
No	92.01 (88.19, 94.67)	86.54 (81.79, 90.20)	80.50 (70.11, 87.91)	
BMI, kg/m^2^	26.77 (26.42, 27.12)	28.56 (27.77, 29.35)	28.58 (27.74, 29.42)	0.001
SBP, mmHg	127.08 (125.86, 128.31)	133.95 (132.20, 135.69)	132.88 (129.77, 135.99)	< 0.001
DBP, mmHg	76.09 (75.24, 76.94)	76.89 (75.82, 77.97)	74.86 (73.18, 76.55)	0.061
TG, mmol/L	1.87 (1.77, 1.97)	1.87 (1.77, 1.98)	1.95 (1.79, 2.11)	0.553
TC, mmol/L	5.47 (5.41, 5.53)	5.75 (5.67, 5.82)	5.71 (5.58, 5.83)	< 0.001
LDL‑C, mmol/L	3.37 (3.31, 3.43)	3.70 (3.58, 3.82)	3.62 (3.41, 3.82)	< 0.001
HDL‑C, mmol/L	1.31 (1.28, 1.34)	1.28 (1.25, 1.31)	1.24 (1.19, 1.30)	0.025
Lp(a), g/L	0.21 (0.19, 0.23)	0.25 (0.22, 0.29)	0.33 (0.28, 0.37)	< 0.001
BUN, mmol/L	5.39 (5.24, 5.54)	5.60 (5.41, 5.80)	6.09 (5.70, 6.48)	0.013
CR, umol/L	97.47 (96.26, 98.68)	99.09 (97.60, 100.59)	105.08 (100.99, 109.18)	0.003
UA, umol/L	323.88 (317.73, 330.03)	330.39 (325.21, 335.56)	337.48 (317.70, 357.27)	0.206
FPG, mmol/L	5.58 (5.51, 5.66)	6.02 (5.82, 6.22)	6.34 (6.11, 6.56)	< 0.001
HbA1c, %	5.50 (5.44, 5.56)	5.84 (5.75, 5.94)	6.03 (5.93, 6.14)	< 0.001
Outcomes, n (%)				
All-cause mortality				< 0.001
Yes	36.54 (33.06, 40.17)	52.48 (46.60, 58.30)	64.36 (54.43, 73.20)	
No	63.46 (59.83, 66.94)	47.52 (41.70, 53.40)	35.64 (26.80, 45.57)	
CVD-related mortality				< 0.001
Yes	12.16 (10.15, 14.50)	19.21 (16.16, 22.67)	25.42 (19.78, 32.02)	
No	87.84 (85.50, 89.85)	80.79 (77.33, 83.84)	74.58 (67.98, 80.22)	
Cancer-related mortality				< 0.001
Yes	7.41 (6.13, 8.94)	12.08 (10.04, 14.47)	13.86 (10.33, 18.34)	
No	92.59 (91.06, 93.87)	87.92 (85.53, 89.96)	86.14 (81.66, 89.67)	

Data were expressed as weighted mean (95% CI), or weighted percentage (95% CI)

*Abbreviation*: *FIB* Fibrinogen, *PIR* Poverty income ratio, *CVD* Cardiovascular disease, *BMI* Body mass index, *SBP* Systolic blood pressure, *DBP* Diastolic blood pressure, *TG* Triglycerides, *TC* Total cholesterol, *LDL-C* Low-density lipoprotein cholesterol, *HDL-C* High-density lipoprotein cholesterol, *Lp(a)* Lipoprotein (a), *BUN* Blood urea nitrogen, *CR* Creatinine, *UA* Uric acid, *FPG* Fasting plasma glucose, *HbA1c* Hemoglobin A1c, *CI* Confidence interval

### Associations between Lp(a) and FIB with mortality

During the total follow-up time of a median of 235 months, 2,668 individuals died, of which 1,051 died of CVD and 549 died of cancer. As shown in Table 3, after adjusting solely for age and sex, higher levels of Lp(a) and FIB, as well as their combination were all associated with increased all-cause, CVD and cancer-related mortality (*P* < 0.05).

**Table 3:** Age and sex adjusted association of Lp(a) and FIB categories with cause-specific mortality

		**All-cause mortality**		**CVD-related mortality**		**Cancer-related mortality**	
		**HR (95% CI)**	***P* value**	**HR (95% CI)**	***P* value**	**HR (95% CI)**	***P* value**
**Lp(a)**	Lp(a)‑L	Reference		Reference		Reference	
	Lp(a)‑M	1.136 (1.047, 1.233)	0.002	1.272 (1.116, 1.449)	< 0.001	1.171 (0.978, 1.402)	0.085
	Lp(a)‑H	1.215 (1.072, 1.377)	0.002	1.487 (1.228, 1.800)	< 0.001	1.328 (1.014, 1.739)	0.039
	Per 1 unit increment	1.304 (1.154, 1.474)	< 0.001	1.655 (1.379, 1.987)	< 0.001	1.383 (1.058, 1.806)	0.018
**FIB**	FIB‑L	Reference		Reference		Reference	
	FIB‑M	1.230 (1.132, 1.337)	< 0.001	1.214 (1.062, 1.388)	0.004	1.431 (1.191, 1.720)	< 0.001
	FIB‑H	1.703 (1.515, 1.914)	< 0.001	1.835 (1.532, 2.199)	< 0.001	1.852 (1.424, 2.407)	< 0.001
	Per 1 unit increment	1.201 (1.149, 1.256)	< 0.001	1.233 (1.150, 1.322)	< 0.001	1.264 (1.152, 1.387)	< 0.001
**Lp(a) + FIB**	Lp(a)‑L + FIB‑L	Reference		Reference		Reference	
	Lp(a)‑L + FIB‑M	1.193 (1.064, 1.337)	0.002	1.248 (1.034, 1.507)	0.021	1.368 (1.063, 1.759)	0.015
	Lp(a)‑L + FIB‑H	1.472 (1.235, 1.755)	< 0.001	1.670 (1.265, 2.204)	< 0.001	1.237 (0.799, 1.917)	0.340
	Lp(a)‑M + FIB‑L	1.090 (0.961, 1.235)	0.179	1.346 (1.102, 1.644)	0.004	1.070 (0.812, 1.410)	0.630
	Lp(a)‑M + FIB‑M	1.323 (1.171, 1.495)	< 0.001	1.431 (1.171, 1.747)	< 0.001	1.476 (1.127, 1.932)	0.005
	Lp(a)‑M + FIB‑H	1.870 (1.565, 2.234)	< 0.001	2.222 (1.686, 2.930)	< 0.001	2.313 (1.589, 3.367)	< 0.001
	Lp(a)‑H + FIB‑L	0.950 (0.755, 1.194)	0.659	1.160 (0.812, 1.658)	0.415	0.878 (0.522, 1.476)	0.623
	Lp(a)‑H + FIB‑M	1.395 (1.162, 1.676)	< 0.001	1.778 (1.345, 2.352)	< 0.001	1.763 (1.200, 2.589)	0.004
	Lp(a)‑H + FIB‑H	2.344 (1.843, 2.980)	< 0.001	3.039 (2.119, 4.357)	< 0.001	2.850 (1.711, 4.748)	< 0.001

Cause-specific HRs and 95% CIs from Cox regression were adjusted for age and sex

*Abbreviation*: *Lp(a)* Lipoprotein (a), *FIB* Fibrinogen, *CVD* Cardiovascular disease, *PIR* Poverty income ratio, *BMI* Body mass index, *SBP* Systolic blood pressure, *DBP* Diastolic blood pressure, *TG* Triglycerides, *TC* Total cholesterol, *LDL-C* Low-density lipoprotein cholesterol, *FPG* Fasting plasma glucose, *HbA1c* Hemoglobin A1c, *CR* Creatinine, *UA* Uric acid, *L* low, *M* Medium, *H* High, *HR* Hazard ratio, *CI* Confidence interval

After adjusting for all confounders (as presented in Table 4), compared with participants with both low Lp(a) and low FIB levels, the multivariable adjusted HRs for all-cause, CVD and cancer-related mortality were the highest for participants with both high Lp(a) and high FIB levels. Compared to the reference group of participants with low Lp(a) and low FIB levels, multivariable adjusted HRs (95% CIs) for all-cause mortality were 1.305 (1.093–1.559) for participants in the Lp(a)-L + FIB-H group, 1.217 (1.074–1.379) for participants in the Lp(a)-M + FIB-M group, 1.615 (1.348–1.935) for participants in the Lp(a)‑M + FIB‑H group, 1.317 (1.090–1.591) for participants in the Lp(a)‑H + FIB‑M group, and 1.813 (1.419–2.317) for participants in the Lp(a)‑H + FIB‑H group, respectively. Similarly, for CVD-related mortality, the HRs (95% CIs) were 1.413 (1.066–1.873) for participants in the Lp(a)-L + FIB-H group, 1.267 (1.033–1.554) for participants in the Lp(a)‑M + FIB‑L group, 1.247 (1.016–1.531) for participants in the Lp(a)‑M + FIB‑M group, 1.874 (1.415–2.482) for participants in the Lp(a)‑M + FIB‑H group, 1.556 (1.165–2.079) for participants in the Lp(a)‑H + FIB‑M group, and 2.147 (1.483–3.109) for participants in the Lp(a)‑H + FIB‑H group, respectively. However, for cancer-related mortality,, only Lp(a)-M + FIB-M, Lp(a)-M + FIB-H, Lp(a)-H + FIB-M and Lp(a)-H + FIB-H groups exhibited statistically significant HRs (95% CIs) compared to the first group.

**Table 4:** Multivariate adjusted association of Lp(a) and FIB categories with cause-specific mortality

		**All-cause mortality**		**CVD-related mortality**		**Cancer-related mortality**	
		**HR (95% CI)**	***P* value**	**HR (95% CI)**	***P* value**	**HR (95% CI)**	***P* value**
**Lp(a)**	Lp(a)‑L	Reference		Reference		Reference	
	Lp(a)‑M	1.124 (1.034, 1.223)	0.006	1.235 (1.080, 1.413)	0.002	1.176 (0.978, 1.414)	0.085
	Lp(a)‑H	1.118 (0.979, 1.276)	0.099	1.237 (1.008, 1.519)	0.042	1.324 (0.999, 1.754)	0.051
	Per 1 unit increment	1.222 (1.073, 1.392)	0.003	1.415 (1.160, 1.725)	0.001	1.389 (1.049, 1.838)	0.022
**FIB**	FIB‑L	Reference		Reference		Reference	
	FIB‑M	1.149 (1.055, 1.251)	0.001	1.117 (0.974, 1.281)	0.115	1.290 (1.070, 1.555)	0.008
	FIB‑H	1.508 (1.338, 1.699)	< 0.001	1.600 (1.330, 1.926)	< 0.001	1.632 (1.248, 2.134)	< 0.001
	Per 1 unit increment	1.148 (1.096, 1.203)	< 0.001	1.175 (1.092, 1.265)	< 0.001	1.212 (1.097, 1.338)	< 0.001
**Lp(a) + FIB**	Lp(a)‑L + FIB‑L	Reference		Reference		Reference	
	Lp(a)‑L + FIB‑M	1.066 (0.949, 1.197)	0.282	1.084 (0.895, 1.313)	0.410	1.207 (0.935, 1.558)	0.148
	Lp(a)‑L + FIB‑H	1.305 (1.093, 1.559)	0.003	1.413 (1.066, 1.873)	0.016	1.117 (0.718, 1.737)	0.624
	Lp(a)‑M + FIB‑L	1.050 (0.925, 1.193)	0.449	1.267 (1.033, 1.554)	0.023	1.072 (0.811, 1.417)	0.626
	Lp(a)‑M + FIB‑M	1.217 (1.074, 1.379)	0.002	1.247 (1.016, 1.531)	0.035	1.346 (1.021, 1.773)	0.035
	Lp(a)‑M + FIB‑H	1.615 (1.348, 1.935)	< 0.001	1.874 (1.415, 2.482)	< 0.001	2.040 (1.390, 2.994)	< 0.001
	Lp(a)‑H + FIB‑L	0.778 (0.611, 0.991)	0.042	0.755 (0.508, 1.121)	0.163	0.855 (0.505, 1.450)	0.561
	Lp(a)‑H + FIB‑M	1.317 (1.090, 1.591)	0.004	1.556 (1.165, 2.079)	0.003	1.718 (1.156, 2.555)	0.007
	Lp(a)‑H + FIB‑H	1.813 (1.419, 2.317)	< 0.001	2.147 (1.483, 3.109)	< 0.001	2.355 (1.396, 3.973)	0.001

HRs and 95% CIs from Cox regression were adjusted for age, sex, race, education, family PIR, ideal exercise, smoking status, drinking, CVD, cancer, diabetes, hypertension, hypercholesterolemia, hypotensive drugs, hypoglycemic drugs, cholesterol-lowering drugs, BMI, SBP, DBP, TG, TC, LDL-C, HDL-C, FPG, HbA1c, and CR

*Abbreviation*: *Lp(a)* lipoprotein (a), *FIB* Fibrinogen, *CVD* Cardiovascular disease, *PIR* Poverty income ratio, *BMI* Body mass index, *SBP* Systolic blood pressure, *DBP* Diastolic blood pressure, *TG* Triglycerides, *TC* Total cholesterol, *LDL-C* Low-density lipoprotein cholesterol, *HDL-C* High-density lipoprotein cholesterol, *FPG* Fasting plasma glucose, *HbA1c* Hemoglobin A1c, *CR* Creatinine, *L* Low, *M* Medium, *H* High, *HR* Hazard ratio, *CI* Confidence interval

**Fig. 2:**
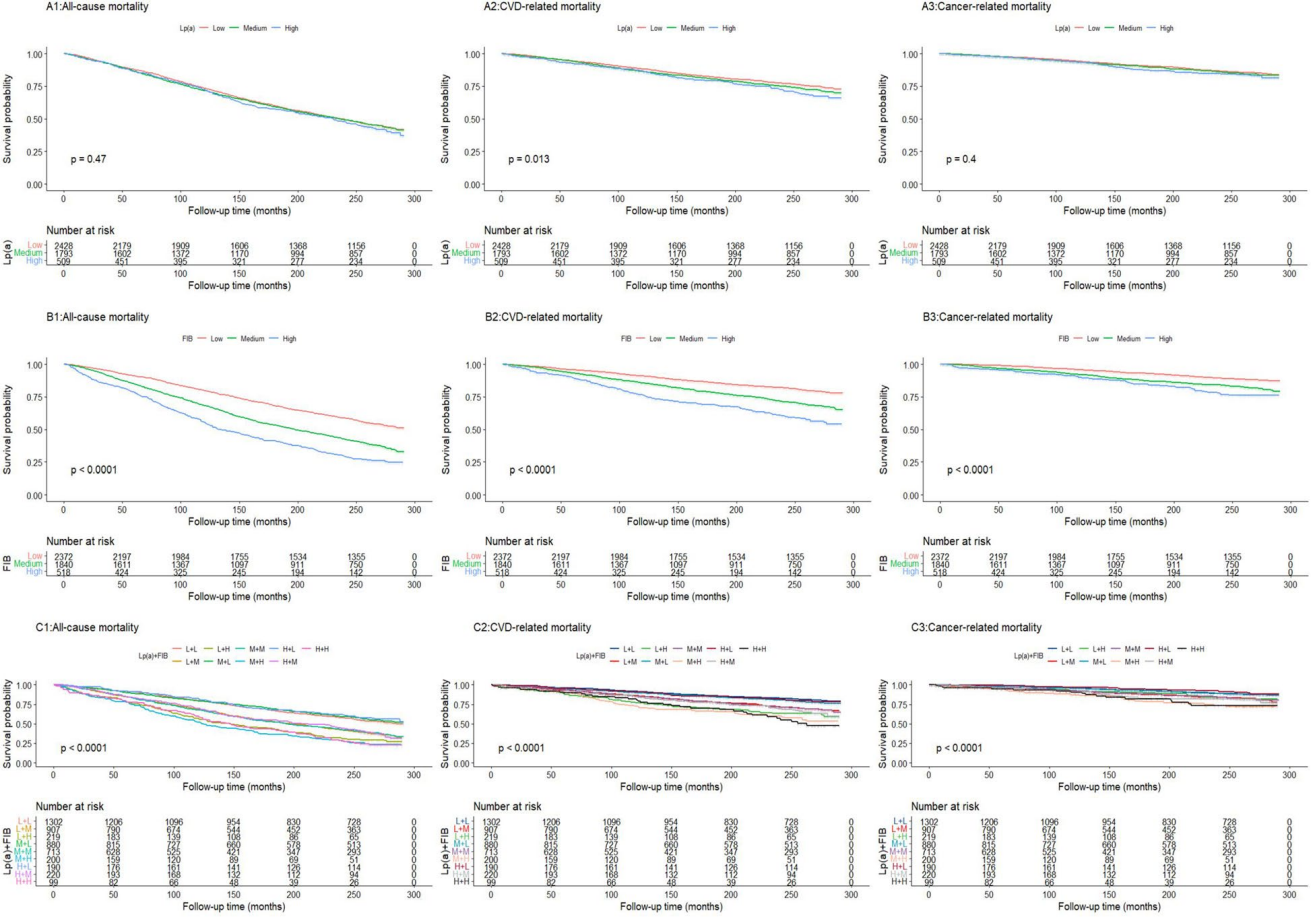
Kaplan–Meier survival curves for (A1, B1, C1) all-cause mortality, (A2, B2, C2) CVD-related mortality, and (A3, B3, C3) cancer-related mortality by Lp(a), FIB and Lp(a) + FIB. CVD, cardiovascular disease; Lp(a), lipoprotein (a); FIB, fibrinogen; L, low; M, medium; H, high

Additionally, as depicted in Fig. 2, the Kaplan–Meier analysis revealed that the variation in survival probability across the three groups was statistically significant solely for CVD-related mortality in relation to Lp(a) (*P* = 0.013). However, for FIB and combined categories, the differences in survival probabilities among the groups were universally significant for all-cause, CVD and cancer-related mortality (*P* < 0.001).

### Subgroup analysis

Table 5 illustrated that in the sex-stratified subgroup analysis, FIB as a continuous variable was associated with the risk of all-cause, CVD and cancer-related mortality in both men and women (*P* < 0.05). Additionally, Lp(a) as a continuous variable was significantly associated with an increased risk of all-cause and CVD-related mortalityin both men and women; however its association with cancer-related mortality was only statistically significant in men (*P* < 0.05). For categorical variables, the risk of all-cause and cancer-related mortality in the Lp(a)-M group was 1.149 and 1.354 times higher in women than in the Lp(a)-L group, respectively. In men, the risk of CVD-related mortality in the Lp(a)-M and Lp(a)-H groups was 1.240 and 1.370 times higher than that in the Lp(a)-L group, respectively (*P* < 0.05). Similarly, women in the FIB-H group experienced a 1.483, 1.509, and 1.697 times higher risk of all-cause, CVD-related, and cancer-related mortality, respectively, compared to those in the FIB-L group (*P* < 0.01). In men, the risk of all-cause, CVD and cancer-related mortality remained higher in the FIB-H group (1.416, 1.470 and 1.503 times, respectively, *P* < 0.05), while the FIB-M group had a higher risk of all-cause and cancer-related mortality only (1.187 and 1.722 times, respectively, *P* < 0.01). For combined categories, among women, compared with the Lp(a)-L + FIB-L group, the risk of all-cause mortality for the Lp(a)-L + FIB-H, Lp(a)-M + FIB-M, Lp(a)-M + FIB-H, and Lp(a)-H + FIB-H groups increased by 1.408, 1.216, 1.600 and 1.670 times, respectively, and the risk of CVD and cancer-related mortality in the Lp(a)-M + FIB-H and Lp(a)-H + FIB-H groups was notably higher, with increases of 1.773 and 1.823 times for CVD-related mortality and 2.352 and 2.385 times for cancer-related mortality, respectively (*P* < 0.05). Among men, with the same reference group, the risk of all-cause CVD, and cancer-related mortality was significantly higher in multiple Lp(a) and FIB combined groups (*P* < 0.05). Additionally, in the subgroup analysis, a significant interaction between FIB and the combination of Lp(a) and FIB with gender was observed, suggesting that the associations between these biomarkers and mortality risks differed significantly between men and women (P for interaction < 0.001).

**Table 5:** Multivariate adjusted association of Lp(a) and FIB categories with mortality by sex

		**Women**	**Men**
		**HR (95% CI)**	**HR (95% CI)**
**All-cause mortality**	Lp(a)‑L	Reference	Reference
	Lp(a)‑M	1.149 (1.021, 1.293)*	1.093 (0.968, 1.233)
	Lp(a)‑H	1.130 (0.946, 1.349)	1.184 (0.970, 1.444)
	P for interaction	0.351	
	Per 1 unit increment	1.247 (1.049, 1.482)*	1.252 (1.026, 1.528)*
	FIB‑L	Reference	Reference
	FIB‑M	1.085 (0.962, 1.223)	1.187 (1.050, 1.342)**
	FIB‑H	1.483 (1.252, 1.758)***	1.416 (1.190, 1.684)***
	P for interaction	0.256	
	Per 1 unit increment	1.147 (1.070, 1.228)***	1.125 (1.054, 1.200)***
	Lp(a)‑L + FIB‑L	Reference	Reference
	Lp(a)‑L + FIB‑M	1.055 (0.895, 1.243)	1.073 (0.910, 1.266)
	Lp(a)‑L + FIB‑H	1.408 (1.089, 1.821)**	1.160 (0.901, 1.494)
	Lp(a)‑M + FIB‑L	1.121 (0.931, 1.350)	1.008 (0.845, 1.202)
	Lp(a)‑M + FIB‑M	1.216 (1.021, 1.449)*	1.207 (1.005, 1.449)*
	Lp(a)‑M + FIB‑H	1.600 (1.225, 2.089)**	1.481 (1.152, 1.904)**
	Lp(a)‑H + FIB‑L	0.927 (0.656, 1.310)	0.770 (0.552, 1.074)
	Lp(a)‑H + FIB‑M	1.153 (0.886, 1.500)	1.569 (1.191, 2.067)**
	Lp(a)‑H + FIB‑H	1.670 (1.236, 2.257)**	2.545 (1.622, 3.994)***
	P for interaction	0.183	
**CVD-related mortality**	Lp(a)‑L	Reference	Reference
	Lp(a)‑M	1.191 (0.987, 1.438)	1.240 (1.021, 1.505)*
	Lp(a)‑H	1.229 (0.936, 1.614)	1.370 (1.006, 1.865)*
	P for interaction	0.766	
	Per 1 unit increment	1.409 (1.082, 1.835)*	1.490 (1.102, 2.015)*
	FIB‑L	Reference	Reference
	FIB‑M	1.075 (0.887, 1.302)	1.083 (0.888, 1.321)
	FIB‑H	1.509 (1.155, 1.973)**	1.470 (1.125, 1.922)**
	P for interaction	0.781	
	Per 1 unit increment	1.183 (1.062, 1.319)**	1.114 (1.004, 1.238)*
	Lp(a)‑L + FIB‑L	Reference	Reference
	Lp(a)‑L + FIB‑M	1.045 (0.800, 1.364)	1.065 (0.806, 1.409)
	Lp(a)‑L + FIB‑H	1.300 (0.855, 1.977)	1.372 (0.923, 2.039)
	Lp(a)‑M + FIB‑L	1.162 (0.861, 1.568)	1.336 (1.009, 1.768)*
	Lp(a)‑M + FIB‑M	1.205 (0.908, 1.599)	1.195 (0.882, 1.620)
	Lp(a)‑M + FIB‑H	1.773 (1.174, 2.678)**	1.662 (1.122, 2.464)*
	Lp(a)‑H + FIB‑L	0.918 (0.531, 1.588)	0.826 (0.484, 1.409)
	Lp(a)‑H + FIB‑M	1.271 (0.852, 1.897)	1.907 (1.245, 2.920)**
	Lp(a)‑H + FIB‑H	1.823 (1.149, 2.894)*	2.855 (1.486, 5.487)**
	P for interaction	0.644	
**Cancer-related mortality**	Lp(a)‑L	Reference	Reference
	Lp(a)‑M	1.354 (1.024, 1.792)*	1.090 (0.850, 1.398)
	Lp(a)‑H	1.387 (0.912, 2.110)	1.378 (0.937, 2.026)
	P for interaction	0.351	
	Per 1 unit increment	1.373 (0.921, 2.047)	1.500 (1.012, 2.222)*
	FIB‑L	Reference	Reference
	FIB‑M	0.929 (0.698, 1.237)	1.722 (1.342, 2.209)***
	FIB‑H	1.697 (1.158, 2.488)**	1.503 (1.025, 2.204)*
	P for interaction	< 0.001	
	Per 1 unit increment	1.181 (1.007, 1.387)*	1.223 (1.076, 1.390)**
	Lp(a)‑L + FIB‑L	Reference	Reference
	Lp(a)‑L + FIB‑M	0.811 (0.546, 1.205)	1.705 (1.218, 2.386)**
	Lp(a)‑L + FIB‑H	0.913 (0.449, 1.858)	1.245 (0.705, 2.198)
	Lp(a)‑M + FIB‑L	1.097 (0.722, 1.667)	1.109 (0.759, 1.620)
	Lp(a)‑M + FIB‑M	1.120 (0.744, 1.687)	1.673 (1.145, 2.445)**
	Lp(a)‑M + FIB‑H	2.352 (1.358, 4.076)**	1.783 (1.032, 3.081)*
	Lp(a)‑H + FIB‑L	0.766 (0.324, 1.810)	0.987 (0.502, 1.939)
	Lp(a)‑H + FIB‑M	1.044 (0.547, 1.992)	2.658 (1.592, 4.438)***
	Lp(a)‑H + FIB‑H	2.385 (1.269, 4.484)**	2.498 (0.878, 7.110)
	P for interaction	< 0.001	

HRs and 95% CIs were adjusted for age, race, education, family PIR, ideal exercise, smoking status, drinking, CVD, cancer, diabetes, hypertension, hypercholesterolemia, hypotensive drugs, hypoglycemic drugs, cholesterol-lowering drugs, BMI, SBP, DBP, TG, TC, LDL-C, HDL-C, FPG, HbA1c, and CR

*Abbreviation*: *Lp(a)* lipoprotein (a), *FIB* Fibrinogen, *CVD* Cardiovascular disease, *PIR* Poverty income ratio, *BMI* Body mass index, *SBP* Systolic blood pressure, *DBP* Diastolic blood pressure, *TG* Triglycerides, *TC* Total cholesterol, *LDL-C* Low-density lipoprotein cholesterol, *HDL-C* High-density lipoprotein cholesterol, *FPG* Fasting plasma glucose, *HbA1c* Hemoglobin A1c, *CR* Creatinine, *L* Low, *M* Medium, *H* High, *HR* Hazard ratio, *CI* Confidence interval

^*^P < 0.05

^**^P < 0.01

^***^P < 0.001

### Sensitivity analysis

In the sensitivity analysis detailed in Table 6, elevated levels of Lp(a) was still closely associated with all-cause and CVD-related mortality after excluding individuals who died within the initial two years of follow-up (*P* < 0.05). Besides, although the association between higher FIB levels and an increased risk of all-cause, CVD, and cancer-related mortality remained consistent with the results presented in Table 4, the HRs and *P* value were attenuated. More importantly, individuals with elevated levels of both Lp(a) and FIB continued to exhibit a significantly heightened risk of all-cause, CVD and cancer-related mortality compared to those with lower levels of both biomarkers (*P* < 0.05).

**Table 6:** Multivariate adjusted association of Lp(a) and FIB categories with mortality after excluding participants who died within two years of follow-up

		**All-cause mortality**		**CVD-related mortality**		**Cancer-related mortality**	
		**HR (95% CI)**	***P* value**	**HR (95% CI)**	***P* value**	**HR (95% CI)**	***P* value**
**Lp(a)**	Lp(a)‑L	Reference		Reference		Reference	
	Lp(a)‑M	1.121 (1.027, 1.224)	0.010	1.224 (1.062, 1.410)	0.005	1.195 (0.987, 1.446)	0.068
	Lp(a)‑H	1.097 (0.956, 1.260)	0.187	1.189 (0.957, 1.477)	0.117	1.265 (0.942, 1.697)	0.118
	Per 1 unit increment	1.182 (1.031, 1.355)	0.017	1.354 (1.097, 1.670)	0.005	1.293 (0.963, 1.735)	0.087
**FIB**	FIB‑L	Reference		Reference		Reference	
	FIB‑M	1.135 (1.039, 1.240)	0.005	1.126 (0.976, 1.300)	0.105	1.222 (1.009, 1.481)	0.040
	FIB‑H	1.406 (1.237, 1.598)	< 0.001	1.515 (1.240, 1.851)	< 0.001	1.360 (1.014, 1.823)	0.040
	Per 1 unit increment	1.117 (1.063, 1.175)	< 0.001	1.143 (1.055, 1.238)	0.001	1.144 (1.027, 1.274)	0.014
**Lp(a) + FIB**	Lp(a)‑L + FIB‑L	Reference		Reference		Reference	
	Lp(a)‑L + FIB‑M	1.060 (0.940, 1.195)	0.342	1.108 (0.909, 1.352)	0.310	1.136 (0.873, 1.477)	0.342
	Lp(a)‑L + FIB‑H	1.215 (1.002, 1.472)	0.047	1.287 (0.944, 1.754)	0.111	0.841 (0.504, 1.403)	0.507
	Lp(a)‑M + FIB‑L	1.053 (0.923, 1.202)	0.443	1.238 (0.998, 1.535)	0.052	1.090 (0.822, 1.444)	0.551
	Lp(a)‑M + FIB‑M	1.200 (1.054, 1.366)	0.006	1.251 (1.010, 1.550)	0.040	1.273 (0.959, 1.691)	0.095
	Lp(a)‑M + FIB‑H	1.544 (1.271, 1.875)	< 0.001	1.871 (1.386, 2.526)	< 0.001	1.879 (1.249, 2.827)	0.002
	Lp(a)‑H + FIB‑L	0.796 (0.621, 1.019)	0.070	0.813 (0.544, 1.215)	0.312	0.802 (0.465, 1.381)	0.425
	Lp(a)‑H + FIB‑M	1.293 (1.062, 1.573)	0.010	1.460 (1.073, 1.986)	0.016	1.666 (1.113, 2.494)	0.013
	Lp(a)‑H + FIB‑H	1.627 (1.248, 2.121)	< 0.001	1.924 (1.285, 2.880)	0.001	1.794 (0.996, 3.233)	0.052

HRs and 95% CIs from Cox regression were adjusted for age, sex, race, education, family PIR, ideal exercise, smoking status, drinking, CVD, cancer, diabetes, hypertension, hypercholesterolemia, hypotensive drugs, hypoglycemic drugs, cholesterol-lowering drugs, BMI, SBP, DBP, TG, TC, LDL-C, HDL-C, FPG, HbA1c, and CR

*Abbreviation*: *Lp(a)* Lipoprotein (a), *FIB* Fibrinogen, *CVD* Cardiovascular disease, *PIR* Poverty income ratio, *BMI* Body mass index, *SBP* Systolic blood pressure, *DBP* Diastolic blood pressure, *TG* Triglycerides, *TC* Total cholesterol, *LDL-C* Low-density lipoprotein cholesterol, *HDL-C* High-density lipoprotein cholesterol, *FPG* Fasting plasma glucose, *HbA1c* Hemoglobin A1c, *CR* Creatinine, *L* Low, *M* Medium, *H* High, *HR* Hazard ratio, *CI* Confidence interval

### RCS analysis

**Fig. 3:**
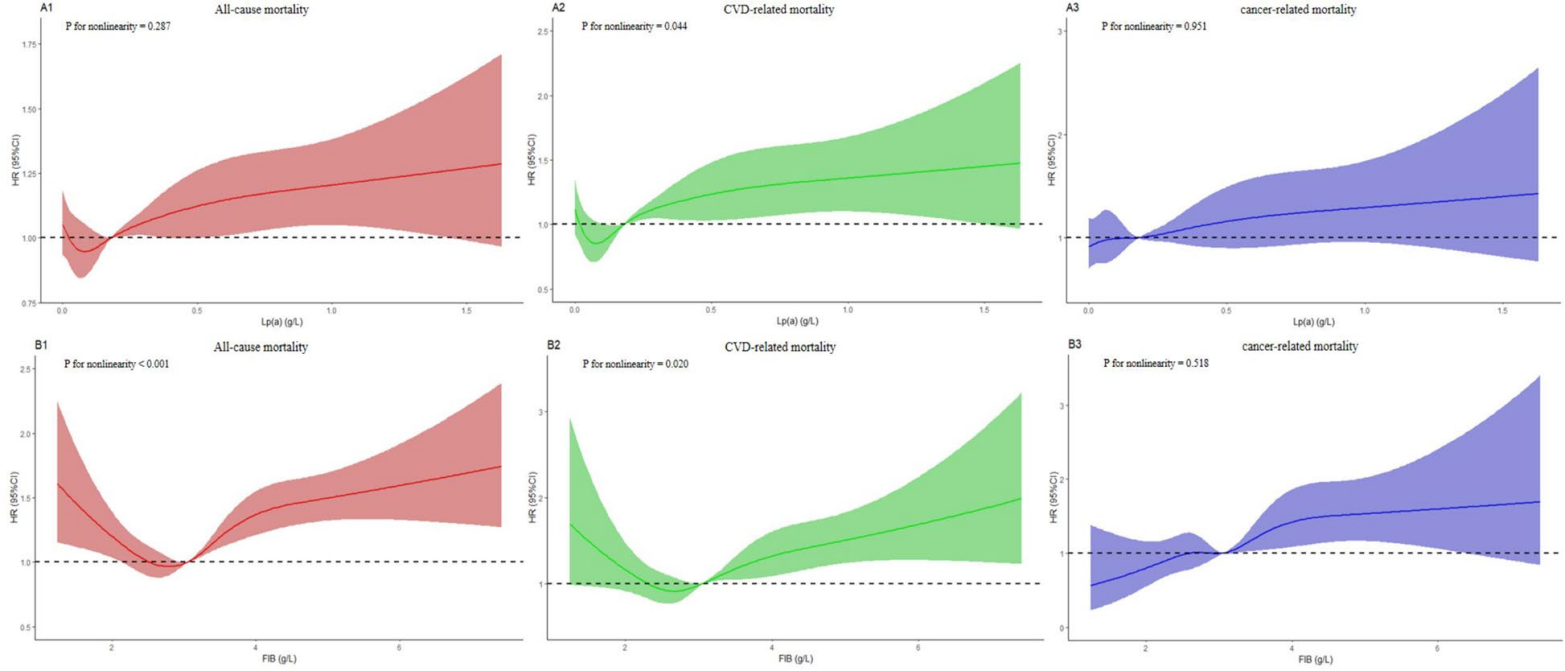
Hazard ratios for all-cause mortality (A1, B1), CVD-related mortality (A2, B2), and cancer-related mortality (A3, B3) according to Lp(a) and FIB. CVD, cardiovascular disease; Lp(a), lipoprotein (a); FIB, fibrinogen

As shown in Fig. 3, the RCS analysis indicated that the association between Lp(a) and the risk of all-cause and cancer-related mortality, as well as the association between FIB and the risk of cancer-related mortality were linear (P for nonlinearity > 0.05), whereas the association between Lp(a) and the risk of CVD-related mortality, as well as the association between FIB and the risk of all-cause and CVD-related mortality were nonlinear (P for nonlinearity < 0.05).

## Discussion

In this large prospective cohort study involving the general population, we found, for the first time, that extremely high levels of both Lp(a) and FIB together conferred a 1.8-fold risk of all-cause mortality, a 2.1-fold risk of CVD-related mortality, and a 2.4-fold risk of cancer-related mortality, suggesting that individuals with both Lp(a) and FIB at higher levels may have a higher risk of mortality. This novel insight underscores the critical importance of monitoring these biomarkers as part of comprehensive health assessments to identify individuals at elevated risk.

Discovered by Berg in 1963, Lp(a) was subsequently identified as a low-density lipoprotein-like particle primarily composed of oxidized phospholipids, apoB, and apo(a), the latter of which is covalently bonded to apoB via disulfide bonds [29]. Originating from the plasminogen gene thousands of years ago, apo(a) shares a degree of structural homology with plasminogen, suggesting potential functional similarities between Lp(a) and the fibrinolytic system [10, 11, 29, 30]. This complex and diverse structure of Lp(a) enables it to play varied roles in promoting atherosclerosis through its low-density lipoprotein components, inflammation, and oxidative stress through oxidized phospholipids, lipid transport via apoB, and thrombosis and antifibrinolysis through apo(a) components, thus establishing its close relationship with the fibrinolytic system [9, 11, 31–34]. FIB, a central component of fibrinolytic system, is a critical factor in thrombosis and antifibrinolysis and has long been recognized as an independent risk factor for thrombosis and CVD [35–38]. In recent years, the growing interest in Lp(a) has led to reports of its association with CVD and thrombosis, including large-scale observational and genetic studies suggesting a causal relationship between Lp(a) and CVD, as well as findings linking Lp(a) to venous thromboembolism [2, 3, 39–42]. Further research has begun to unveil functional similarities between Lp(a) and FIB, with studies confirming Lp(a)'s affinity for protease-modified FIB through its lysine binding site structure, providing a theoretical foundation for exploring the correlation between Lp(a) and FIB [43]. Subsequently, Ganotakis et al. found that there was a significant positive correlation between Lp(a) and FIB in patients with primary hyperlipidemia, but this correlation was affected by gender, smoking status and complications [44]. In view of this, we hypothesized that there may be synergistic effects of Lp(a) and FIB in certain diseases as previous studies have also explored. For example, in a prospective cohort study involving 2,125 adults without coronary heart disease, Cantin et al. found that individuals with FIB levels higher than 4.05 g/L and Lp(a) levels higher than 300 mg/L had a higher risk of coronary heart disease compared to individuals with FIB levels lower than 4.05 g/L and Lp(a) levels lower than 300 mg/L. Furthermore, a study by Zhang et al. showed that the risk of cardiovascular events in patients with both high Lp(a) and FIB was 2.656 times higher than in patients with both low Lp(a) and FIB [45]. Since atherosclerosis and thrombosis, which are closely related to Lp(a) and FIB [46], are also independent risk factors for mortality, we hypothesized that Lp(a) and FIB may also have additive or synergistic effects on mortality. This hypothesis was explored as early as 2006 by D'Angelo et al., who found that high Lp(a) combined with high FIB was associated the occurrence of coronary heart disease or stroke-related death without finding an independent association of Lp(a) or FIB alone with mortality [47]. However, the study was limited by its small sample size and the fact that the outcome variable was restricted to coronary heart disease or stroke-related death. In addition, although there is a substantial body of literature reporting their association with CVD and mortality, our study attempted to explore this topic from a new angle, namely by analyzing the combined effects of Lp(a) and FIB on all-cause, CVD, and cancer-related mortality. We believe that this cross-classification approach can provide new insights into the potential differential impacts of these biomarkers in combination at different levels on mortality risk. Therefore, to comprehensively explore the association of the combination of Lp(a) and FIB with all-cause, CVD, and cancer-related mortality, we conducted this study and found that high levels of both Lp(a) and FIB together conferred a 1.8-fold risk of all-cause mortality, a 2.1-fold risk of CVD-related mortality, and a 2.4-fold risk of cancer-related mortality compared with low levels of both Lp(a) and FIB. Additionally, several other studies also have also corroborated the association of Lp(a) or FIB with mortality [4, 5, 12, 13, 15–17, 48, 49]. However, we are not sure whether simultaneous reduction of Lp(a) and FIB can reduce the risk of mortality in this high-risk population, which needs to be evaluated by further studies and clinical trials. Additionally, while these findings provide foundational data for potentially developing predictive models in the future, the actual assessment of predictive ability necessitates further specialized research for validation.

Despite the important findings, our study had several limitations. First, we failed to establish the causal association of Lp(a) or FIB, or their combined categories, with mortality due to the observational nature of our study. Second, because Lp(a) and FIB were only measured once at baseline, we were unable to assess the impact of their dynamic changes on mortality during the follow-up period. Third, we may not have been able to control for all potential confounding variables because of the large number of factors that affect mortality. Fourth, our comparison of individuals who were simultaneously in the high percentiles for both Lp(a) and FIB with those in the low percentiles may not have fully considered the potential impact of each biomarker in extreme groups, potentially leading to an overinterpretation of the combined effects of Lp(a) and FIB. Therefore, to address this issue, we plan to employ more rigorous statistical methods in future research to assess the independent and interactive effects of Lp(a) and FIB on mortality risk. This may involve using multivariable models to simultaneously consider the levels of Lp(a) and FIB and evaluate their interactions with mortality risk, as well as considering more complex statistical methods to explore the impact of individual biomarkers in more extreme groups. Moreover, we also believed that merely restricting the population to extreme cases with both high Lp(a) and high FIB did not directly lead to the conclusion that this group had a higher risk compared to groups selected based on a single biomarker. Fifth, this study only covered 14% of the original population based on the NHANES study, so the result may not be representative. But we believe that despite the reduced sample size, such stringent screening could enhance the credibility and applicability of our study conclusions, and through strict inclusion and exclusion criteria and careful control of potential biases, we believe our study results could provide strong insights into the associations between Lp(a), FIB, and their combination with all-cause, CVD, and cancer-related mortality. For future research directions, we suggest using a broader sample coverage and exploring other potential biomarkers to further validate and expand our findings. Sixth, although there appeared to be no significant correlation between Lp(a) and outcomes in the full adjusted model, there was a combined effect of Lp(a) and FIB on the probability of mortalities, making it difficult to determine whether the affection may bring on by FIB alone. Therefore, We recognize that there may be limitations in analyzing the independent and combined effects of these variables. For example, there might be other confounding factors not considered, or the sample size might not be sufficient to reveal more complex relationships, so we suggest that future research should consider using larger sample sizes and more complex statistical methods to further explore the combined effects of these biomarkers. Seventh, due to the crossing phenomenon in several Kaplan–Meier survival analysis curve diagrams, which is similar to other studies above, we cannot determine whether this phenomenon is caused by Lp(a) or does not conform to the proportional hazard hypothesis. We will further explore these issues in future research. Finally, although the mechanisms through which Lp(a) and FIB are associated with mortality have been extensively studied, the underlying mechanisms behind the combined impact of Lp(a) and FIB on mortality remain unknown. Therefore, further research is needed in the future to uncover the potential mechanisms involved..

## Conclusions

In this cohort study, we presented novel findings showing that, in comparison to categorizing Lp(a), FIB, or any other combined metrics individually, extremely high levels of both Lp(a) and FIB concurrently were associated with a significantly increased risk of mortality from all-cause, CVD, and cancer, indicating that individuals exhibiting elevated levels of both Lp(a) and FIB warrant more vigilant monitoring and the implementation of more intensive risk management strategies. The aim is to mitigate the heightened risk of premature death and reduce overall mortality rates among this high-risk group..

## Data Availability

All of the data and materials used in this study can be accessed free of charge on the publicly available NHANES official website (https://www.cdc.gov/nchs/index.htm).
